# Unexpected depletion in plasma choline and phosphatidylcholine concentrations in a pregnant woman with bipolar affective disorder being treated with lithuim, haloperidol and benztropine: a case report

**DOI:** 10.1186/1752-1947-2-55

**Published:** 2008-02-20

**Authors:** Maxine Gossell-Williams, Horace Fletcher, Steven H Zeisel

**Affiliations:** 1 Department of Basic Medical Sciences, University of the West Indies, Jamaica; 2 Department of Obstetrics, Gynaecology and Child Health, University of The West Indies, Jamaica; 3 Department of Nutrition, School of Public Health and School of Medicine University of North Carolina at Chapel Hill, NC 27599, USA

## Abstract

**Introduction:**

Patients with bipolar affective disorder can be effectively managed with pharmacological intervention. This case report describes a pregnant woman with a ten-year history of bipolar affective disorder that was being treated with lithium, haloperidol and benztropine.

**Case presentation:**

The patient had a normal pregnancy, but developed an elevated blood pressure and started to lose weight at 36 weeks of gestation. During pregnancy, plasma concentrations of choline and phosphatidylcholine are increased to meet the demands of the foetus. However, our findings in this case included depletion of plasma choline and phosphatidylcholine concentrations. Other unusual outcomes included low placental weight and low infant birth weight.

**Conclusion:**

This report suggests that the pharmacological management of this patient could possibly account for the findings.

## Introduction

Choline is a nutrient that is a precursor of phosphatidylcholine and the plasma concentrations of both nutrients are controlled by endogenous synthesis and dietary intake [1]. Both are important for the efficient turnover of lipids from the liver and blood. Choline is also important for the control of plasma homocysteine concentration and is the precursor of the neurotransmitter acetylcholine, which is important for the proper functioning of cholinergic neurons peripherally and in the brain.

Patients with bipolar affective disorder are effectively managed with pharmacological intervention, such as lithium, haloperidol and benztropine, but studies on the influence of these drugs on plasma choline and phosphatidylcholine concentrations are limited. There is evidence that lithium can decrease the plasma availability of these important cell components [2,3], but whether this translates into depletion in the brain supply remains questionable [4].

When women with bipolar affective disorder become pregnant, pharmacological management is complicated because of possible risks to the foetus from the use of medications. Lithium, for example is classified as a category D drug [5], that is, having the potential to cause foetal malformations, including foetal cardiac malformations [6,7]. However in the case of pregnant patients with affective disorder, the benefits of therapy can outweigh the risks. We report on the pregnancy outcomes of a patient with bipolar affective disorder treated with mood stabilizers in the antenatal clinic of the University Hospital of the West Indies.

## Case presentation

The patient was a 25-year-old gravida 2, presenting to the antenatal clinic at 13 weeks gestational age. Medical history indicated that the patient was diagnosed with bipolar disorder ten years prior to this pregnancy. She was effectively managed with lithium carbonate (500 mg b.i.d.), haloperidol (Haldol^®^5 mg b.i.d.) and benztropine (Cogentin^®^, 2 mg b.i.d.) prior to pregnancy and the regimen was continued through the pregnancy. Plasma concentrations of the prescribed medication were not assessed. At 12 weeks gestation, the patient described her appetite as good, with two full meals and three snacks per day. Her pregnancy booking BMI was 21.99 and plasma haemoglobin (Hb) was normal (11.4 mg/dL). Her haemoglobin phenotype status is AA and she was both HIV and VDRL negative. The patient also reported regular supplementation with multivitamins specific for pregnancy (Materna^®^). Her blood pressure was normal at the beginning of the pregnancy at 110/70 mmHg and remained normal until about 36 weeks of gestation. The weight gain from 15 to 36 weeks of gestation was 6.9 Kg and her Hb remained in the normal range throughout the pregnancy. She then started to lose weight moving from 73.1 Kg at 36 weeks (+ 5 days) to 71.4 Kg at 38 weeks (+ 5 days) and recorded an elevation in blood pressure from week 37 until week 38. Her blood studies at 37 weeks were all normal (Table 1).

**Table 1:** Haematological indexes measured for bipolar affective disorder patient

Sodium	135 mmol/l	Globulin	31 g/l
Potassium	4.7 mmol/l	Direct Bilirubin	7 umol/l
Urea	1.9 mmol/l	Total Bilirubin	22 umol/l
Creatinine	33 umol/ml	Alkaline Phosphotase	85 IU/l
Uric acid	0.18 mmol/l	G.G.T.	7 IU/l
Total protein	63 mmol/l	S.G.O.T.	31 IU/l
Albumin	32 g/l	PT	13.8/12.6
		PTT	32.8/30.6

She was admitted to the antenatal ward at 38 weeks + 5 days and labour was induced, however due to failure to progress, a caesarean section was performed with the birth of a male infant at 39 weeks. The infant's birth Apgar scores were good: 9 at one minute and 10 at 5 minutes. The infant's birth weight was 2500 g, which is below the mean for a term baby in the Jamaican population [8]. Both infant and mother were discharged after three days and no follow-up data of either was collected.

The patient in this study was taken from a pool of sixteen women who were followed through all three trimesters of pregnancy. In order to make further assessment of infant outcomes in this case, we selected other women from the larger study that were similarly matched in gestation age, weight gain, blood pressure, haemoglobin status and infant gestational age at birth (Table 2). The most distinctive differences between these other women and this patient were the lower birth weight of the infant (30% less) and lower placental weight (42% less).

**Table 2:** Comparison of variables between the patient with bipolar affective disorder and control patients.

Variable	**Bipolar patient**.	Means ± S.D. N = 3
Age/yrs	25	29 ± 9
Height/cm	173.5	164.8 ± 7.2
BMI	22	26.8 ± 2.3
Weight Gain/Kg	6.9	6.5 ± 1.0
13 weeks Systolic/mmHg	100	103 ± 15
22 weeks Systolic/mmHg	100	120 ± 10
36 weeks Systolic/mmHg	110	110 ± 10
13 weeks Diastolic/mmHg	60	63 ± 6
22 weeks Diastolic/mmHg	60	73 ± 6
36 weeks Diastolic/mmHg	80	77 ± 15
1^st ^trimester Hb (g/dl)	11.4	14.2 ± 3.7
2^nd ^trimester Hb (g/dl)	11.4	11.4 ± 1.1
3^rd ^trimester Hb (g/dl)	11.9	11.2 ± 0.3
Parity	2	2 ± 2
Gestational age (days)	272	273 ± 2
Birth weight (g)	2500	3573 ± 133
Placental weight (g)	350	607 ± 51
Crown Heel length (cm)	51	48.5 ± 3.0
Head Circumference (cm)	32	33.6 ± 0.7
Ponderal index (g/cm3)	18.8	32 ± 7.1
Head Circumference:length ratio	62.7	69.5 ± 3.0
Placenta: Birth weight ratio	14	17 ± 0.8

The patient with bipolar affective disorder had lower infant birth weight and lower placental weight, resulting in lower ponderal index and placenta:birthweight ratio.

We measured both fasting plasma phosphatidylcholine and choline through the three trimesters of pregnancy (Table 3). For this patient, comparison between the data from trimester 1 (week 10–13) to trimester 3 (week 34–37) showed that plasma phosphatidylcholine concentration decreased by 22% during this period, while the plasma choline decreased by 38%. Comparison of the controls showed the expected increase in plasma choline and phosphatidylcholine concentrations.

**Table 3:** Plasma phosphatidylcholine and choline concentration.

		Bipolar patient	Means ± S.D. N = 3
**PHOSPHATIDYLCHOLINE (nmoles/ml)**			
	10–13 weeks	2158.96	1573.24 ± 50.73
	19–23 weeks	2180.34	1769.70 ± 324.59
	34–37 weeks	1677.86	1716.11 ± 423.70
**FREE CHOLINE(nmoles/ml)**			
	10–13 weeks	11.17	8.94 ± 1.81
	19–23 weeks	7.62	8.77 ± 1.48
	34–37 weeks	6.89	10.99 ± 1.94

The table shows the plasma concentrations of phospahtidylcholine and choline for trimester 1 (10–13 weeks gestation), trimester 2 (19–23 weeks gestation) and trimester 3 (34–37 weeks gestation) for bipolar and control patients. The patient with bipolar affective disorder showed depletion of both compounds rather than the expected increase.

## Conclusion

We found that in the case of our patient, there was an unusually low placental weight and a low infant birth weight when compared with data recorded from three control patients and from previous studies of our population [8]. These previous studies also recorded an association of low birth weight infants with low haemoglobin concentrations, especially during the first trimester. However, this was not a factor in this case, as the patient maintained normal plasma concentrations of haemoglobin throughout the pregnancy.

Low weight gain during pregnancy is another risk factor that contributes to low infant birth weight [9]; however, the control patients that experienced similar weight gain did not give birth to low birth weight infants. Although our comparisons are limited by the lack of dietary intake information, previous reports have confirmed that mood stabilizers can contribute to low birth weight outcome [10].

On further comparison of this patient with controls, it appeared that there was a decrease in plasma choline and phosphatidylcholine concentrations in this patient. Both nutrients are especially important during pregnancy and are actively transported to the foetus [1,11]. The decreases in plasma concentrations of these nutrients in our patient were unexpected, as plasma concentrations of both are increased during pregnancy [12,13], possibly to ensure adequate supply to the foetus. Phosphatidylcholine, for example, supplies important long chain polyunsaturated fatty acids, and deficiency of polyunsaturated fatty acids to the foetus is a known risk factor for negative foetal outcomes such as low birth weight [14]. Furthermore, animal studies have demonstrated that inadequate maternal supply of these nutrients impairs cognitive and memory functions of pups and that dietary supplementation with these nutrients during pregnancy can prevent these effects [1,15].

Our data analysis was limited by the lack of information on the actual amounts of choline and phosphatidylcholine that were consumed by this patient during pregnancy and therefore whether inadequate dietary intake contributed to the unexpected depletions. However, previously documented evidence supports negative influences of at least one of the drugs involved (lithium) on these nutrients. We therefore conclude that there is need for further studies to clarify the causal associations between drug therapy, maternal outcomes, foetal outcomes and the availability of these nutrients in patients being treated for bipolar affective disorder. Whether benefits could be derived from dietary supplementation with choline and phosphatidylcholine should also be considered.

## Competing interests

The author(s) declare that they have no competing interests.

## Authors' contributions

SZ acted as principal investigator on the study and was responsible for the assessment of the maternal data. HF acted as obstetric/gynaecology consultant and assessed the maternal outcomes. MG was the investigator responsible for the collection data and overall assessment. All authors have read and approved this manuscript.

## Consent

Signed written informed consent was received from all patients reported in this paper allowing for publication of the data. A copy of the written consent is available for review by the editor-in-chief of this journal.
